# Body mass index and hospital mortality in mechanically ventilated patients with acute hypoxaemic respiratory failure: A retrospective cohort study

**DOI:** 10.1016/j.ccrj.2026.100216

**Published:** 2026-09-08

**Authors:** Philip Emerson, Peiyao Du, Helena Oakey, Momina Allahwala, Krish Sundararajan, Mark P. Plummer

**Affiliations:** a The Royal Adelaide Hospital, Department of Intensive Care Medicine, Adelaide, South Australia, Australia; b Guys and St Thomas' NHS Foundation Trust, London, United Kingdom; c South Australian Health and Medical Research Institute, Department of Biostatistics, Adelaide, South Australia, Australia; d Lyell McEwin Hospital, Adelaide, South Australia, Australia; e Adelaide University, Adelaide, South Australia, Australia

**Keywords:** Acute hypoxaemic respiratory failure, Obesity, Body mass index, Mechanical ventilation

## Abstract

**Background:**

An obesity paradox has been described in general and selected intensive care populations, whereby obese patients experience lower mortality than healthy-weight patients despite physiological disadvantages. Whether this paradox exists among a selected group of patients with acute hypoxaemic respiratory failure (AHRF) requiring mechanical ventilation remains uncertain. We examined the association between body mass index (BMI) and in-hospital mortality in a large, contemporary cohort of mechanically ventilated intensive care unit (ICU) patients with AHRF.

**Methods:**

This retrospective cohort study used the ANZICS Adult Patient Database (2020–2024). We included unplanned adult ICU admissions requiring invasive mechanical ventilation with AHRF, defined as admission PaO_2_/FiO_2_ <300 mm Hg. The primary outcome was in-hospital mortality, assessed using hierarchical logistic regression after adjustment for confounders. The nonlinear association between respiratory failure severity and mortality was modelled using restricted cubic splines. The secondary outcomes included organ support use, tracheostomy receipt, duration of ventilation, and ICU and hospital length of stay.

**Results:**

Among 40 352 eligible patients, overall unadjusted in-hospital mortality was 21% (22.7% in healthy-weight patients). After adjustment, mortality was lower in preobese patients (BMI 25–29.9 kg/m^2^; adjusted odds ratio [aOR] 0.92, 95% confidence interval [CI] 0.85–0.99), obesity class 1 (BMI 30–34.9 kg/m^2^; aOR 0.85, 95% CI 0.79–0.93), and obesity class 2 (BMI 35–39.9 kg/m^2^; aOR 0.88, 95% CI 0.79–0.98). The association weakened after adjustment and did not extend to obesity class 3 (BMI ≥40 kg/m^2^). There was no significant interaction between BMI and respiratory failure severity (p = 0.08).

**Conclusion:**

Among patients ventilated for AHRF, overweight and moderately obese BMI categories were associated with modestly lower in-hospital mortality. This association is conditional on the decision to intubate and is compatible with a modest degree of selection bias. The conclusions should be interpreted as a prognostic association within ventilated patients rather than the presence of obesity improving survival.

## 1 Introduction

Acute hypoxaemic respiratory failure (AHRF) accounts for 52% of intensive care unit (ICU) admissions in Australia and New Zealand, with a large proportion of affected patients requiring mechanical ventilation.^1^ AHRF is associated with substantial resource utilisation and has an attributable mortality as high as 35%.^1^^,^^2^

Obesity, defined by a body mass index (BMI) ≥30 kg/m^2^, affects one-third of Australian adults,^3^ with the prevalence projected to increase globally over the coming decades.^4^ The interaction between obesity as described by elevated BMI and outcomes in patients with AHRF remains incompletely characterised. From a physiological perspective, obesity is associated with several factors that may plausibly worsen outcomes in hypoxaemic respiratory failure. Increased adipose tissue mass reduces chest wall compliance, elevates intra-abdominal pressure, and impairs diaphragmatic excursion.^5^ These factors predispose obese patients to respiratory decompensation^6^ and would be expected to confer an increased risk of morbidity and mortality once respiratory failure develops.^7^^,^^8^

Paradoxically, multiple observational studies in ICU populations have reported similar or lower mortality among overweight and obese patients compared with those of a healthy weight, a finding referred to as the “obesity paradox”.[8], [9], [10], [11] Proposed explanations include greater metabolic reserves, altered inflammatory and immune responses, and adaptive cardiovascular changes.^5^ However, an alternative explanation is that the observed paradox reflects residual confounding and bias inherent to observational research, including collider stratification bias, survival bias, and incomplete adjustment for illness severity and treatment limitation.^11^^,^^12^ Whether the obesity paradox persists within more clinically homogeneous populations remains uncertain.

Previous studies evaluating the obesity paradox in the ICU setting have been relatively unselective in their inclusion criteria, resulting in a heterogeneous case mix and associated limitations regarding application of the results to specific patient cohorts,^10^ such as those with AHRF. Based on this, we restricted our cohort to a single, clinically recognisable group of adults already receiving invasive ventilation with a confirmed diagnosis of AHRF.

In this paper, we examined the association between BMI category and in-hospital mortality in a highly selected cohort of critically ill patients with AHRF requiring invasive mechanical ventilation at ICU admission. By restricting the cohort to patients already intubated, we aimed to reduce selection bias related to thresholds for ICU admission and intubation and examine the association between BMI and outcomes. We hypothesised that obesity would be associated with a higher in-hospital mortality compared with healthy-weight patients. Additionally, we explored whether this association varied according to the severity of respiratory failure.

## 2 Methods

### 2.1 Ethical approval

The Central Adelaide Local Health Network Ethics Committee approved this study with the need for informed consent waived. The study adhered to Strengthening the Reporting of Observational Studies in Epidemiology (STROBE) guidelines^13^ for observational research and principles outlined in the Declaration of Helsinki.

### 2.2 Study design and participants

This was a retrospective cohort study using the Australia New Zealand Intensive Care Society Adult Patient Database (ANZICS APD).^14^ We included adult patients (aged ≥18 years) admitted to participating ICUs between January 1, 2020, and December 31, 2024, who had a documented height and weight, required mechanical ventilation, and had AHRF defined as PaO_2_/FiO_2_ ratio <300 mm Hg. The lowest PaO_2_ and highest FiO_2_ in the first 24 h of the ICU admission were used to produce the PaO_2_/FiO_2_ ratio, consistent with other studies using this metric.^1^ We excluded readmissions during the same hospital stay, elective surgical admissions, patients with treatment limitations documented at ICU admission, pregnant patients, and those with BMI values below the 1st or above the 99th percentile of the eligible cohort, to avoid values resulting from erroneous registry data entry.

### 2.3 Data sources and definitions

The ANZICS APD is a binational registry collecting data from 98% of Australian and 67% of New Zealand adult ICUs.^14^ Data quality is maintained through standardised data dictionaries, quality assurance processes, and formalised training. The registry collects demographic data, chronic health conditions, physiologic variables from the first 24 h of ICU stay, organ support interventions, and hospital outcomes.

BMI was calculated from height and weight measured at ICU admission and categorised according to World Health Organization criteria:^15^ underweight (<18.5 kg/m^2^), healthy weight (18.5–24.9 kg/m^2^), preobese (25–29.9 kg/m^2^), obese class 1 (30–34.9 kg/m^2^), obese class 2 (35–39.9 kg/m^2^), and obese class 3 (≥40 kg/m^2^). Respiratory failure severity was treated as a continuous variable.

Primary admission diagnosis was classified using the Acute Physiology and Chronic Health Evaluation III (APACHE III) diagnostic system and dichotomised as respiratory versus nonrespiratory. Frailty was assessed using the Clinical Frailty Scale and categorised as not frail (1–3), moderately frail (4–6), or severely frail (7–9).^16^

### 2.4 Statistical analysis

We summarised continuous variables as medians and interquartile ranges, categorical variables as numbers with percentages.

The primary outcome was hospital mortality. The secondary outcomes were discharge destination (home, rehabilitation facility), ICU mortality, ICU and hospital length of stay (hours), duration of mechanical ventilation (hours), receipt of extracorporeal membrane oxygenation (ECMO), and tracheostomy placement. These secondary outcomes were analysed descriptively.

The primary analysis was a hierarchical logistic regression with hospital site specified as random intercepts to evaluate the association between BMI category and in-hospital mortality. Covariates were selected a priori using a directed acyclic graph based on established clinical and pathophysiological relationships to identify a minimally sufficient adjustment set and reduce bias from confounding and inappropriate conditioning^17^ (Supplementary Fig. S1). This model is reported in Table 2 and was prespecified. Models were adjusted for the Australia and New Zealand Risk of Death (ANZROD) score,^18^ age, sex, admission diagnosis (respiratory versus nonrespiratory), hospital type, and Clinical Frailty Score category. Healthy BMI served as the reference category throughout. Analyses were restricted to hospitals contributing more than 50 admissions to ensure adequate estimation of random effects.

We assessed effect modification by AHRF severity by incorporating interaction terms between BMI category and the PaO_2_/FiO_2_ ratio modelled using cubic splines with four degrees of freedom. This approach was used due to the previously reported nonlinear relationship between P:F ratio and mortality in large ANZICS APD cohorts.^1^ Model fit was compared between specifications with and without interaction terms using likelihood ratio tests (LRTs), the Akaike information criterion, and Bayesian information criterion. Our original protocol set statistical significance at p < 0.001. On review, this threshold was too strict for categorical BMI comparisons and was changed to a conventional p < 0.05. We note this as a post hoc change, and interpretation rests primarily on the effect estimates and 95% confidence intervals rather than on a threshold. All analyses were performed using R version 4.5.1 (The R Foundation, Vienna, Austria).^19^ Anonymised analysis code is provided in the Supplementary material.

As the study period included the COVID-19 pandemic, during which obesity was a recognised risk factor for ICU admission,^20^ we performed a sensitivity analysis to determine whether the association between BMI and mortality differed by COVID-19 status. Confirmed COVID-19–positive patients were identified from the APD, and a BMI category by COVID-19 interaction was added to the primary model. The interaction was assessed by an LRT.

Complete case analysis was prespecified as the primary approach. A missing data analysis was performed, establishing that BMI had substantial missingness. To characterise the nature of the missingness, we compared eligible patients with and without recorded BMI on baseline characteristics and crude mortality, confined to the eligible population. We then performed two sensitivity analyses. First, a multiple imputation by chained equations was performed under a missing-at-random assumption.^21^ Estimates were pooled across 40 imputed datasets via Rubin's rules. Second, a tipping point analysis^22^ was performed to evaluate whether BMI data were not missing at random. This was done by progressively shifting the imputed values away from the missing-at-random expectation and assessing how results were affected.

A quantitative bias assessment was performed to estimate the extent that conditioning on intubation could account for our findings. This was done by calculating a selection-bias E-value for each BMI category, as described by Mathur et al.^23^ A selection-bias E-value is the smallest association (on a risk ratio scale) that unaccounted for selection drivers would need to have with BMI and mortality to explain our results in full.^24^

## 3 Results

### 3.1 Patient characteristics

**Fig. 1:**
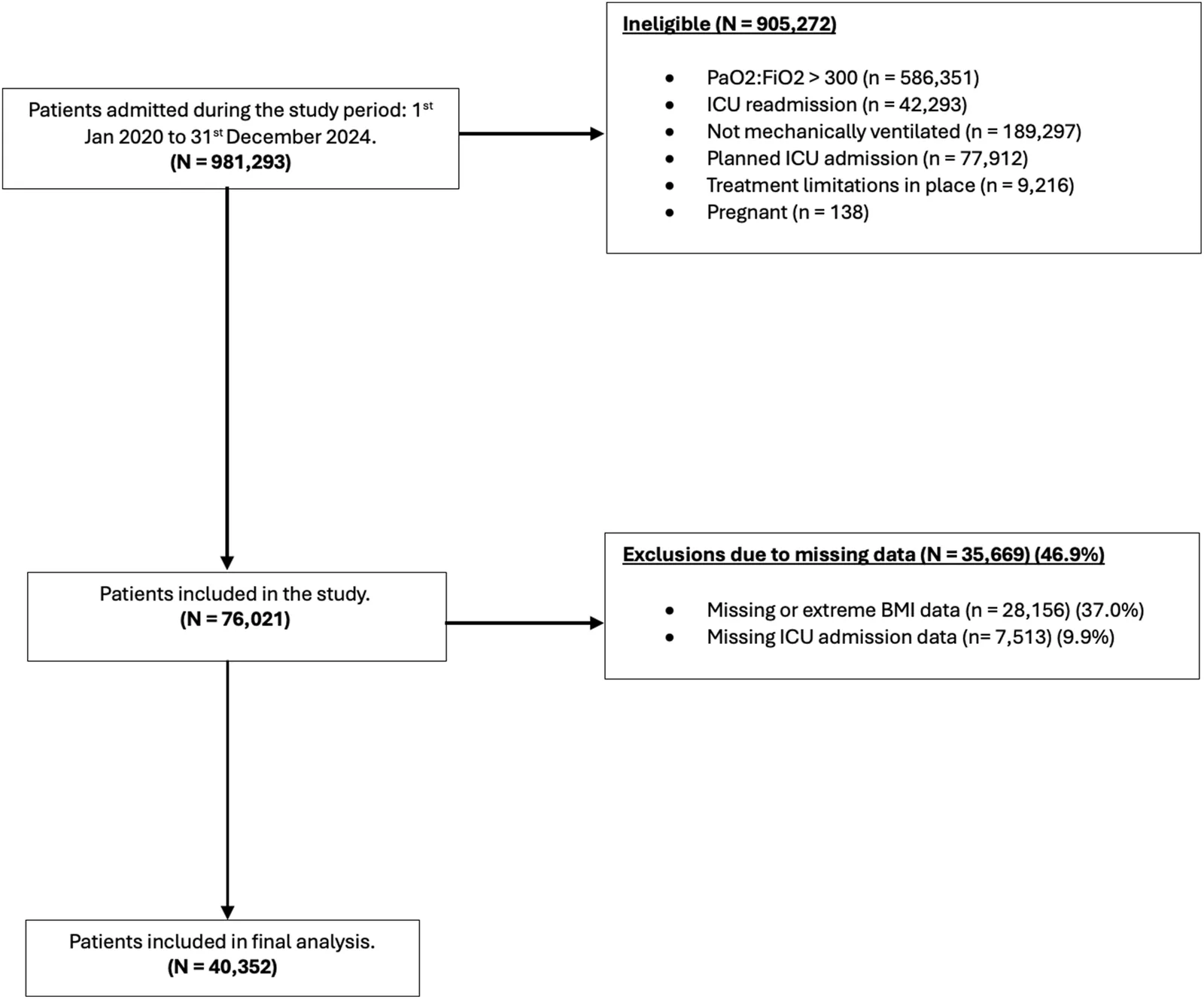
Derivation of study cohort.

**Fig. 2:**
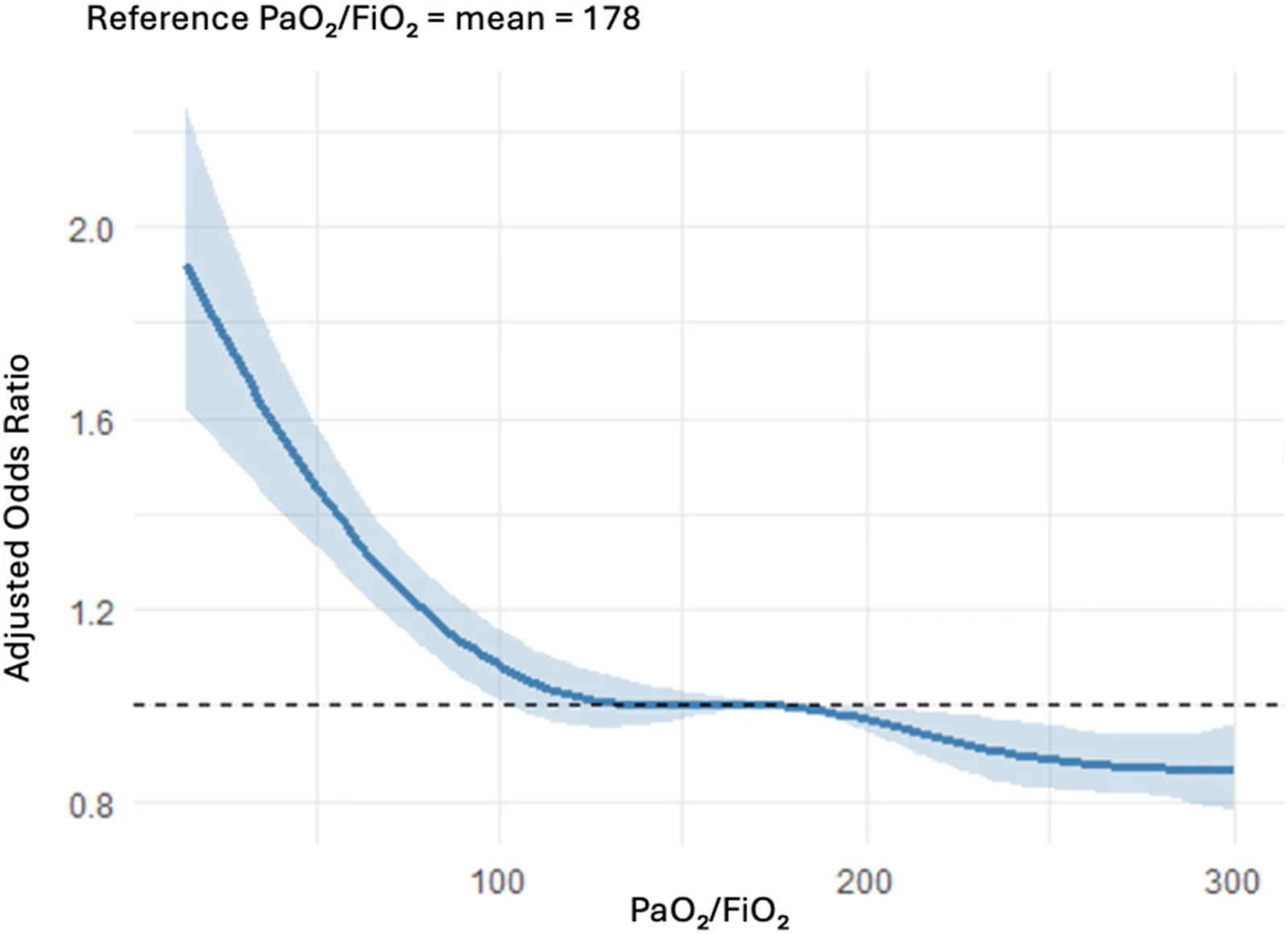
Relationship between PaO_2_/FiO_2_ ratio and hospital mortality. Cubic spline curve showing adjusted odds ratio for hospital death across the spectrum of respiratory failure severity. Reference PaO_2_/FiO_2_ = population mean of 178 mm Hg. Shaded area represents 95% confidence interval. Model adjusted for body mass index category, age, sex, admission diagnosis, hospital type, and frailty category.

Fig. 1 outlines the derivation of the study population. Between January 2020 and December 2024, there were 981 293 adult ICU admissions recorded in the ANZICS APD. Of these, 76 021 met eligibility criteria. Complete BMI data were unavailable for 28 156 (37.0%). A further 7513 (9.9%) were excluded for missing covariate data, giving 35 669 (46.9%) excluded overall, and leaving 40 352 (53.1%) in the analysis cohort (see Fig. 2).

Baseline characteristics stratified by BMI category are presented in Table 1. The median age was 60 (interquartile range 47–70) years, and 36% of patients were female. BMI distribution was 2% underweight, 25% healthy weight, 30% preobese, 21% obese class 1, 11% obese class 2, and 11% obese class 3. The median ANZROD score was 10 (3–31), and the median PaO_2_/FiO_2_ ratio was 177 (121–237) mm Hg.

**Table 1:** Descriptive characteristics by BMI category.

Characteristic	Underweight (n = 719)	Healthy weight (n = 10 112)	Preobese (n = 12 177)	Obese class 1 (n = 8283)	Obese class 2 (n = 4479)	Obese class 3 (n = 4582)	Overall (n = 40 352)
Age, median [Q1, Q3]	56 [40, 67]	59 [43, 70]	62 [48, 72]	62 [50, 71]	59 [48, 69]	55 [45, 65]	60 [47, 70]
Female, n (%)	320 (45)	3687 (36)	3834 (31)	2832 (34)	1723 (38)	2277 (50)	14 673 (36)
**Country,** n (%)							
Australia	645 (90)	8664 (86)	10 521 (86)	7013 (85)	3768 (84)	3790 (83)	34 401 (85)
New Zealand	74 (10)	1448 (14)	1656 (14)	1270 (15)	711 (16)	792 (17)	5951 (15)
**Pre-existing chronic comorbidities,** n (%)							
Cardiovascular	29 (4.0)	398 (3.9)	657 (5.4)	483 (5.8)	268 (6.0)	324 (7.1)	2159 (5.4)
Respiratory	84 (12)	695 (6.9)	660 (5.4)	490 (5.9)	307 (6.9)	511 (11)	2747 (6.8)
Cirrhosis	21 (2.9)	352 (3.5)	329 (2.7)	258 (3.1)	117 (2.6)	110 (2.4)	1187 (2.9)
Dialysis	18 (2.5)	298 (2.9)	330 (2.7)	203 (2.5)	140 (3.1)	122 (2.7)	1111 (2.8)
Immunosuppressed	32 (4.5)	393 (3.9)	363 (3.0)	233 (2.8)	109 (2.4)	95 (2.1)	1225 (3.0)
**Disease severity**, median [Q1, Q3]							
Apache III score	76 [58, 94]	73 [54, 94]	70 [52, 92]	69 [51, 92]	66 [48, 88]	63 [46, 85]	69 [51, 92]
ANZROD score	14 [5, 33]	12 [4, 35]	11 [3, 33]	10 [3, 31]	9 [3, 27]	7 [2, 21]	10 [3, 31]
PaO_2_/FiO_2_ ratio	179 [124, 243]	185 [125, 246]	183 [124, 242]	174 [121, 233]	170 [116, 229]	160 [111, 220]	177 [121, 237]
**Primary diagnosis,** n (%)							
Nonrespiratory	541 (75)	8273 (82)	10 167 (83)	6914 (83)	3678 (82)	3526 (77)	33 099 (82)
Respiratory	178 (25)	1839 (18)	2010 (17)	1369 (17)	801 (18)	1056 (23)	7253 (18)
**Hospital type,** n (%)							
Private	16 (2.2)	464 (4.6)	708 (5.8)	490 (5.9)	248 (5.5)	215 (4.7)	2141 (5.3)
Public metropolitan	159 (22)	2102 (21)	2394 (20)	1726 (21)	981 (22)	1217 (27)	8579 (21)
Public regional	119 (17)	1220 (12)	1482 (12)	1054 (13)	628 (14)	658 (14)	5161 (13)
Public tertiary	425 (59)	6326 (63)	7593 (62)	5013 (61)	2622 (59)	2492 (54)	24 471 (61)
**Clinical frailty score,** n (%)							
1-3	306 (43)	5936 (59)	7776 (64)	5183 (63)	2685 (60)	2376 (52)	24 262 (60)
4-6	362 (50)	3880 (38)	4136 (34)	2925 (35)	1698 (38)	2055 (45)	15 056 (37)
7-8	51 (7.1)	296 (2.9)	265 (2.2)	175 (2.1)	96 (2.1)	151 (3.3)	1034 (2.6)
Delirium during ICU stay	111 (18)	1711 (20)	2032 (20)	1210 (18)	686 (18)	626 (16)	6376 (19)
**ICU interventions**							
Tracheostomy**,** n (%)	38 (5.3)	566 (5.6)	660 (5.5)	404 (4.9)	222 (5.0)	216 (4.8)	2106 (5.3)
ECMO**,** n (%)	7 (1.0)	237 (2.4)	278 (2.3)	174 (2.1)	95 (2.1)	71 (1.6)	862 (2.2)
Vasopressor support	610 (85)	8398 (83)	9994 (82)	6738 (82)	3633 (81)	3567 (78)	32 940 (82)
Dialysis	78 (11)	1473 (15)	1846 (15)	1301 (16)	755 (17)	749 (17)	6202 (16)
Duration of ventilation, median [Q1, Q3]	50 [19, 125]	52 [18, 136]	51 [18, 137]	50 [17, 136]	53 [17, 149]	52 [18, 141]	51 [18, 138]
**Hospital outcome,** n (%)							
Death	176 (25)	2277 (23)	2581 (21)	1637 (20)	823 (18)	772 (17)	8266 (21)
Home	354 (49)	4902 (49)	6070 (50)	4243 (51)	2310 (52)	2430 (53)	20 309 (50)
Inter-hospital transfer	99 (14)	1567 (16)	1905 (16)	1354 (16)	741 (17)	776 (17)	6442 (16)
Nursing home	10 (1.4)	175 (1.7)	188 (1.5)	116 (1.4)	58 (1.3)	66 (1.4)	613 (1.5)
Rehabilitation	56 (7.8)	878 (8.7)	1102 (9.1)	695 (8.4)	399 (8.9)	392 (8.6)	3522 (8.7)
**Length of stay**, median [Q1, Q3]							
ICU (hours)	117 [58, 231]	112 [52, 225]	113 [51, 231]	112 [50, 228]	116 [52, 239]	114 [52, 238]	113 [51, 231]
Hospital (hours)	285 [126, 568]	282 [129, 553]	281 [133, 549]	280 [134, 526]	288 [139, 544]	287 [147, 554]	283 [134, 546]

ANZROD, Australia and New Zealand Risk of Death Score; ECMO, extracorporal membrane oxygenation; ICU, intensive care unit; PaO_2_/FiO_2_, partial pressure of arterial oxygen to inspired oxygen ratio.

**Table 2:** Hospital mortality among patients intubated with acute hypoxaemic respiratory failure by BMI category.

BMI category	Adjusted OR (95% CI)	p-value
Underweight	1.10 (0.90–1.36)	0.35
Normal	1.00 (ref)	–
Preobese	0.92 (0.85–0.99)	0.03
Obese class 1	0.85 (0.79–0.93)	<0.001
Obese class 2	0.88 (0.79–0.97)	0.01
Obese class 3	0.92 (0.82–1.02)	0.10

BMI, body mass index; OR, odds ratio; ref, reference.

Adjusted for age, sex, ANZROD risk of death, hospital type, and frailty category.

Mixed-effects logistic regression with hospital as random effect.

Reference: Normal BMI (18.5–24.9 kg/m^2^).

Compared with healthy-weight patients, those in obese categories were younger, more frequently female, and had lower illness severity scores (APACHE III and ANZROD). The prevalence of chronic respiratory disease exhibited a U-shaped distribution across BMI categories, being highest among underweight (17%) and obese class 3 (17%) patients and lowest among healthy-weight patients (9.6%). A similar U-shaped pattern was observed for frailty, with the highest prevalence of frailty in underweight patients (54%) and the lowest in obese class 1 (41%) patients.

With regard to the BMI missing data analysis, 64 779 of the 76 021 analytical cohort had complete covariate data for imputation analysis. Eligible patients with and without recorded BMI were similar, including crude mortality (22% vs 21%; Supplementary Table S1).

### 3.2 Primary outcome: hospital mortality

Overall in-hospital mortality was 21%. Unadjusted mortality differed across BMI categories, decreasing progressively with greater BMI: 24.5% in underweight patients, 22.7% in healthy-weight patients, 21.4% in preobese patients, 20.1% in obesity class 1, 18.8% in obesity class 2, and 17.2% in obesity class 3 (Supplementary Table S2 and Fig. S2).

In the hierarchical mixed-effects logistic regression adjusted for ANZROD, age, sex, primary diagnosis, hospital type, and Clinical Frailty Score, BMI category was significantly associated with in-hospital mortality (Table 2). Compared with healthy-weight patients, underweight patients had a numerically greater but not statistically significant increased risk of death (odds ratio [OR] 1.10, 95% confidence interval [CI] 0.90–1.36, p = 0.35). In contrast, higher BMI categories were associated with modestly reduced adjusted mortality, including preobese patients (OR 0.92, 95% CI 0.85–0.99, p = 0.03), obesity class 1 (OR 0.85, 95% CI 0.79–0.93, p < 0.001), obesity class 2 (OR 0.88, 95% CI 0.79–0.97, p < 0.01). Obesity class 3 was not significantly associated with reduced mortality (OR 0.92, 95%CI 0.82–1.02, p = 0.10).

Covariate missingness was minimal, with 1.9% missing Clinical Frailty Score data and near-complete data for all other covariates. Supplementary Table S3 describes patients with and without Clinical Frailty Score data.

Under multiple imputation, the association between BMI and hospital mortality was essentially unchanged (Supplementary Table S4). In the tipping point analysis, the association between obesity categories and lower mortality persisted across shifts of up to 3 kg/m^2^ in the imputed values, with only marginal attenuation at the largest shifts (Supplementary Table S5).

Selection bias E-values, as part of the quantitative bias assessment, are reported in Supplementary Table S6.

### 3.3 Effect modification by respiratory failure severity

Mortality risk increased steeply as PaO_2_/FiO_2_ ratio decreased below approximately 150 mm Hg, with odds of death exceeding twofold at PaO_2_/FiO_2_ ratios below 80 mm Hg relative to the cohort mean ratio of 178 mm Hg. In contrast, mortality risk decreased modestly at ratios above 200 mm Hg.

Interaction testing between BMI category and PaO_2_/FiO_2_ ratio demonstrated no evidence of effect modification (LRT p = 0.08; Supplementary Table S7). Consistent with this finding, models excluding interaction terms had lower Akaike information criterion and Bayesian information criterion values, indicating superior fit compared with simpler models.

### 3.4 COVID-19 sensitivity analysis

A total of 1291 participants (3.2%) tested positive for COVID-19 during their ICU admission. There was no evidence that the association between BMI category and hospital mortality differed by COVID-19 status (interaction LRT p = 0.55). A similar pattern of lower mortality among patients in the obese categories was observed in both COVID-19–positive and COVID-19–negative patients, consistent with findings in the main analytical cohort (Supplementary Tables S8 and 9).

### 3.5 Secondary outcomes

Secondary outcomes are summarised descriptively in Table 1 and Supplementary Table S10. Duration of mechanical ventilation, ICU length of stay, hospital length of stay, and the use of ICU interventions other than ECMO were broadly similar across BMI categories, without any clinically meaningful differences observed.

Among 862 ECMO recipients, crude in-hospital mortality was 32% (Supplementary Table S11) and ranged from 23% in obese class 3 to 43% in obese class 1, without the gradient seen in the primary analysis. The low frequency of both ECMO and other interventions, such as tracheostomy, and renal replacement therapy across BMI strata precluded meaningful interpretation of differences in crude rates for these interventions.

## 4 Discussion

In this large binational cohort of mechanically ventilated patients with AHRF, higher BMI was associated with lower in-hospital mortality. The crude difference was substantial, with mortality falling from 24.5% in underweight patients to 17.2% in the most obese group, but most of this difference disappeared after adjustment for illness severity, frailty and other confounders. The adjusted ORs were modest, and for the highest BMI category, the association was no longer statistically significant.

Our findings are consistent with prior observational studies reporting lower or similar mortality among overweight and obese patients in general ICU populations.^10^^,^^25^^,^^26^ We extend these observations to a highly selected cohort of critically ill patients admitted emergently to ICU, already receiving invasive mechanical ventilation for AHRF. This population is notable because obesity is associated with adverse respiratory mechanics, increased work of breathing and impaired gas exchange, factors that would be expected to worsen outcomes in severe respiratory failure.^5^

Multiple biological mechanisms for the “obesity paradox” have been proposed, including greater metabolic reserve, and altered inflammatory response.^8^ However, there is increasing recognition that much of the apparent protective effect of obesity in observational studies may reflect residual confounding and methodological bias rather than a true biological advantage.^11^^,^^12^^,^^27^^,^^28^ Notably, Decruyenaere et al^11^ demonstrated that when machine learning approaches are used to better account for complex confounding, the obesity paradox is attenuated, although not completely eliminated when compared with traditional regression models.

By restricting the cohort to unplanned ICU admissions in patients who were already intubated for AHRF, with no treatment limitations, we hoped to isolate a more homogenous group of patients increasingly encountered in clinical practice. By adjusting for illness severity, frailty, and admission diagnosis using a prespecified causal framework, we sought to minimise some biases that limited previous observational studies.

We acknowledge that significant index-event bias remains and have quantified it via our quantitative bias assessment. This assessment reported small E-values, which are consistent with selection bias existing within our data. In the BMI category with the strongest association (class 1), an unmeasured determinant of intubation associated with both BMI and mortality at a risk ratio of 1.4 would move the CI to the null. This is a plausible effect size, and we are open to the fact that this selection bias could account for our findings in full.

Our estimates were secure against assumptions of missing data (imputation and tipping point analysis) but are not robust against unmeasured confounding that contributes to the intubation decision. Our findings are most appropriately interpreted as a prognostic association within ventilated patients, not as evidence that BMI changes the outcome of patients who are mechanically ventilated with AHRF.

### 4.1 Clinical implications

While acknowledging the influence of residual bias, our findings have important clinical implications. First, obese patients represent a substantial proportion of critically ill individuals requiring invasive ventilation for AHRF, accounting for a large burden of ICU admissions, resource utilisation, and mortality. This underscores the importance of improving both observational and mechanistic research into obesity and respiratory failure to better understand care processes, patient selection, and optimal management strategies in this growing population.^5^^,^^7^

Our findings do not support using BMI to guide the decision to intubate. That decision precedes entry to our cohort, and an association measured after intubation cannot inform it without repeating the same bias. At most, our data suggest that once a patient is ventilated, higher BMI is not a marker of worse outcome. Specifically, our data do not support the position that obesity is protective in AHRF.

Within the limits of our descriptive secondary analyses, the broadly similar use of resource-intensive and invasive interventions, including ECMO, tracheostomy, and renal replacement therapy, across high BMI categories further supports the notion that BMI alone is not driving decisions around intensity of care. This is pertinent, given emerging evidence that frailty is a stronger prognostic determinant than BMI in critically ill patients^16^ and that BMI may not adversely influence outcomes following venovenous ECMO.^29^

Our crude ECMO hospital mortality rates reflect that seen in the international literature.^30^ We note that the pattern of decreasing crude mortality as BMI increases is not seen in the ECMO cohort. Interpreting these ECMO data should be done cautiously, given the lack of data granularity in the APD with regard to ECMO. Specific registries such as the EXCEL^31^ are better placed to evaluate this association.

### 4.2 Strengths and limitations

This study represents the largest observational analysis to date examining the relationship between BMI and mortality in mechanically ventilated patients with respiratory failure, utilising a high-quality registry across two countries.^14^ The restriction to a more clinically homogeneous population strengthens internal validity, an approach increasingly emphasised in critical care research.^32^ Adjustment for frailty is a further strength, given growing recognition of its prognostic importance in the critically ill.^16^ Our methodology is robust and reproducible, with a COVID-19 sensitivity analysis, and a missing data analysis supporting a complete case analysis with regard to BMI.

Several limitations warrant consideration. As an observational study, our findings describe associations rather than causation and may not be generalisable beyond similar health care settings. Residual confounding is likely despite adjustment for multiple clinically relevant variables. Unmeasured factors such as clinician practice variation (particularly the unknown frequency of prone positioning), premorbid nutritional status, unrecorded comorbidities, and body fat distribution may have influenced outcomes.

We recognise that restricting the cohort to patients who were intubated may introduce selection bias because BMI may influence the likelihood of receiving intubation (Supplementary Fig. S1). This cohort selection strategy aimed to isolate the association between BMI and hospital outcome while reducing bias from treatment limitation decisions and differences in the threshold for intubation. However, this introduces index-event bias, such as BMI being correlated with unmeasured determinants of survival such as physiological reserve and pre-ICU course.^11^^,^^12^ Accordingly, our results should be interpreted as describing the prognostic association between BMI and outcome among intubated patients, rather than demonstrating a causal effect of obesity.

We performed a quantitative bias assessment, as described by Mathur et al.,^23^ which was developed to assess the extent to which collider selection bias could explain obesity paradox findings. The resulting selection-bias E-values were small, suggesting that our described association is not robust to selection bias. An unmeasured determinant of intubation of limited strength could account for the associations we observed. This is the principal limitation of the study.

BMI is an imperfect measure of adiposity,^33^ a limitation that is amplified in critical illness where acute fluid shifts can affect weight measurements.^34^ Weight was recorded at admission only, and inaccuracy and/or estimation cannot be excluded. Excluding BMI values beyond the 1st and 99th percentiles may also have removed some genuine patients at the extremes of the distribution rather than only erroneous entries. These patients were not lost entirely because their BMI was treated as missing and imputed in the sensitivity analysis, which were stable. Finally, the secondary outcomes, including ECMO data, were analysed descriptively, and low event rates and lack of data granularity limit interpretability.

## 5 Conclusion

In this large binational cohort of mechanically ventilated patients with AHRF, higher BMI was associated with modestly lower in-hospital mortality. The association weakened after adjustment and is compatible with a modest degree of selection bias arising from the decision to intubate. We interpret these data as a prognostic association within ventilated patients with AHRF rather than evidence of a protective effect of obesity.

## Consent for publication

All co-authors have reviewed the manuscript and consent to the publication of this research paper.

## Funding

This research did not receive any specific grant from funding agencies in the public, commercial, or not-for-profit sectors.

## CRediT authorship contribution statement

All authors met requirements for authorship. Conceptualisation: PE, PD, MA, HO, Methodology, PE, MP, KS. Formal analsysis: PE, PD and HO. Writing - Original draft: PE and MA. Writing - Review and Editing: MP, KS and MA. Supervision: MP, KS and HO.

## Conflict of interest

Senior author, M.P., is an associate editor of Critical Care & Resuscitation. Given his role as associate editor of CC&R, M.P. had no involvement in the peer review of this article and had no access to information regarding its peer review. Full responsibility for the editorial process for this article was delegated to another journal editor. If there are other authors, they declare that they have no known competing financial interests or personal relationships that could have appeared to influence the work reported in this paper.
