# Supplementary material for: Body mass index and hospital mortality in mechanically ventilated patients with acute hypoxaemic respiratory failure: A retrospective cohort study
Source: Crit Care Resusc. 2026 Sep 8;28(4):100216. doi: 10.1016/j.ccrj.2026.100216 (PMC13579679; doi:10.1016/j.ccrj.2026.100216)
Supplement: Supplementary file 1 [file mmc1.pdf]

Supplementary Material: Analytical Code

BMI and Hospital Mortality in Acute Hypoxaemic Respiratory Failure — an Analysis of the ANZICS Adult Patient Database

Analytical code · 2 July 2026

> The dataset was obtained from the ANZICS Adult Patient Database under a data access agreement and cannot be shared publicly. Investigators with approved access can reproduce these analyses using their own extract.

1. Packages

```
library(dplyr)
library(tidyr)
library(ggplot2)
library(lme4)
library(splines)
library(emmeans)
library(broom.mixed)
library(stringr)
library(forcats)
library(mice)
library(flextable)
library(gtsummary)
library(gt)
```

2. Data preparation

2.1 Load data

```
# apd <- read.csv("your_extract.csv")

apd <- apd %>% filter(IcuAdmitYYYY != 2025)
apd <- apd %>% mutate(id = row_number())
```

2.2 Derived variables

```
apd <- apd %>% mutate(

  AP3DIAG = ifelse(AP3DIAG == 0, NA, AP3DIAG),

  AP3diag_binary = ifelse(
    AP3DIAG >= 201 & AP3DIAG <= 213,
    "Respiratory diagnoses",
    "Other diagnoses"
  ),
```

```r
  hosp_type = case_when(
    HospitalClassification == "Metropolitan"     ~ "Public Metropolitan",
    HospitalClassification == "Rural / Regional" ~ "Public Rural / Regional",
    HospitalClassification == "Tertiary"         ~ "Public Tertiary",
    HospitalClassification == "Private"          ~ "Private"
  ),

  SEX    = na_if(SEX, ""),
  Female = ifelse(SEX == "F", 1, 0),

  ANZRODRiskOfDeath = 100 * ANZRODRiskOfDeath,

  # COVID-19 status for Section 8. PANDEMIC == 1 is a confirmed case;
  # everything else, NA included, counts as not positive.
  covid = factor(
    if_else(!is.na(PANDEMIC) & PANDEMIC == 1, "COVID-19", "Not COVID-19"),
    levels = c("Not COVID-19", "COVID-19")
  ),

  FRAILTY_cat = case_when(
    FRAILTY >= 1 & FRAILTY <= 3 ~ "1-3",
    FRAILTY >= 4 & FRAILTY <= 6 ~ "4-6",
    FRAILTY >= 7 & FRAILTY <= 8 ~ "7-8"
  ),

  PA_FI_ratio = PAO2_ANZ / FIO2_ANZ,

  IND_TYPE = case_when(
    INV_IND == 1 ~ 1,
    NIV_IND == 1 ~ 2
  ),

  HEIGHT_m = HEIGHT / 100,
  BMI      = WEIGHT / (HEIGHT_m ^ 2),
  BMICAT   = case_when(
    BMI >= 40       ~ "Obese Class 3",
    BMI >= 35       ~ "Obese Class 2",
    BMI >= 30       ~ "Obese Class 1",
    BMI >= 25       ~ "Pre Obese",
    BMI >= 18.5     ~ "Normal",
    !is.na(BMI)     ~ "Underweight"
  ),
  BMICAT = factor(BMICAT, levels = c(
    "Underweight", "Normal", "Pre Obese",
    "Obese Class 1", "Obese Class 2", "Obese Class 3"
  )),

  HOSP_OUTCM_grp = case_when(
    HOSP_OUTCM == 2                ~ "Death",
    HOSP_OUTCM == 3                ~ "Home",
    HOSP_OUTCM == 4                ~ "Nursing home/Chronic care/Palliative care",
    HOSP_OUTCM %in% c(8, 9, 10) ~ "Other",
    HOSP_OUTCM %in% c(5, 6)     ~ "Inter-hospital transfer",
    HOSP_OUTCM == 7                ~ "Rehabilitation",
    TRUE                           ~ NA_character_
  )
)
```

3. Inclusion and exclusion

```
 total  <- nrow(apd)
step1  <- apd %>% filter(AdmEpisode %in% c(0, 1))
step2  <- step1 %>% filter((PAO2_ANZ / FIO2_ANZ) <= 300)
step3  <- step2 %>% filter(INV_IND == 1 | NIV_IND == 1)
step4  <- step3 %>% filter(PLAN_ICU != 1)
step5  <- step4 %>% filter(TREAT_LMT != 1)
step6  <- step5[step5$PREG_STAT != 1 | is.na(step5$PREG_STAT), ]
step7  <- step6 %>% filter(SEX != "I")

BMI_lower <- quantile(step7$BMI, 0.01, na.rm = TRUE)
BMI_upper <- quantile(step7$BMI, 0.99, na.rm = TRUE)
step8 <- step7 %>%
  filter(!if_any(c(HEIGHT, WEIGHT), is.na)) %>%
  filter(BMI > BMI_lower & BMI < BMI_upper)

step10 <- step8 %>%
  filter(!if_any(c(AGE, SEX, ANZRODRiskOfDeath, AP3diag_binary,
                   HospitalClassification, FRAILTY), is.na))

apd_final <- step10
```

4. Missing data analysis

4.1 Patients with vs without a Clinical Frailty Scale score (Table S5)

```
 apd_compare_FRAILTY <- apd %>%
  mutate(FRAILTY_known = case_when(
    id %in% apd_final$id ~ "Patients with CFS",
    is.na(FRAILTY)       ~ "Patients without CFS",
    TRUE                 ~ NA_character_
  )) %>%
  filter(!is.na(FRAILTY_known))

apd_compare_FRAILTY %>%
  tbl_summary(
    by      = FRAILTY_known,
    include = c(AGE, SEX, BMICAT, AP3diag_binary, ANZRODRiskOfDeath, hosp_type,
                HOSP_OUTCM_grp, INV_HOURS, ICU_HRS, HOSP_HRS,
                TRACHE_IND, ECMO_IND),
    label   = list(
      AGE               ~ "Age",
      SEX               ~ "Sex",
      BMICAT            ~ "BMI category",
      AP3diag_binary    ~ "ICU diagnosis",
      ANZRODRiskOfDeath ~ "ANZROD risk of death (%)",
      hosp_type         ~ "Hospital type",
      HOSP_OUTCM_grp    ~ "Hospital outcome",
      INV_HOURS         ~ "Duration of mechanical ventilation (hours)",
      ICU_HRS           ~ "ICU length of stay (hours)",
      HOSP_HRS          ~ "Hospital length of stay (hours)",
      TRACHE_IND        ~ "Tracheostomy",
      ECMO_IND          ~ "ECMO support"
```

```
    ),
    statistic = list(all_continuous() ~ "{median} [{p25}, {p75}]"),
    missing   = "no"
  ) %>%
  add_overall(last = TRUE)
```

4.2 Missing BMI: like-for-like comparison, MAR imputation, and MNAR tipping-point (Tables S1–S3)

```
# Uses the eligible cohort (step7), which still holds the BMI-missing patients
# the complete-case analysis drops.
# Heavy: this fits glmer many times. Run with PILOT <- TRUE first, then FALSE.

stopifnot(exists("step7"), exists("BMI_lower"), exists("BMI_upper"))

PILOT <- FALSE                      # TRUE = quick check; FALSE = full analysis
m          <- if (PILOT)  5 else 40
delta_grid <- if (PILOT) c(-2, 0, 2) else c(-3, -2, -1, 0, 1, 2, 3)
z <- qnorm(0.975)

fmt_or <- function(est, lo, hi) sprintf("%.2f (%.2f-%.2f)", est, lo, hi)

# ---- 0. Build the eligible dataset (BMI is the only missing variable) -------
# Implausible BMI (outside the 1st/99th trim) -> NA, so it is imputed rather
# than used; this keeps the observed-BMI definition the same as the primary analysis.
elig <- step7 %>%
  filter(!if_any(c(AGE, SEX, ANZRODRiskOfDeath, AP3diag_binary,
                  hosp_type, FRAILTY_cat, PA_FI_ratio, HOSP_OUTCM_grp), is.na)) %>%
  mutate(
    BMI        = ifelse(!is.na(BMI) & (BMI <= BMI_lower | BMI >= BMI_upper), NA, BMI),
    hosp_death = factor(ifelse(HOSP_OUTCM_grp == "Death", 1, 0)),
    SEX        = factor(SEX),
    AP3diag_binary = factor(AP3diag_binary),
    hosp_type  = factor(hosp_type),
    FRAILTY_cat = factor(FRAILTY_cat),
    DSITEID    = factor(DSITEID),
    BMI_missing = ifelse(is.na(BMI), "BMI missing", "BMI recorded")
  )

elig$AGE_scaled               <- as.numeric(scale(elig$AGE))
elig$ANZRODRiskOfDeath_scaled <- as.numeric(scale(elig$ANZRODRiskOfDeath))

cat("Eligible patients (complete covariates): ", nrow(elig), "\n")
cat("  Height missing: ", sum(is.na(step7$HEIGHT)), "\n")
cat("  Weight missing: ", sum(is.na(step7$WEIGHT)), "\n")
cat("  BMI missing/implausible -> imputed: ", sum(is.na(elig$BMI)),
    sprintf(" (%.1f%%)\n", 100 * mean(is.na(elig$BMI))))

# ---- 1. Table S1: like-for-like within the eligible population --------------
# BMI-recorded vs BMI-missing on characteristics and crude mortality, within
# the eligible population (replaces the earlier whole-database comparison).
tbl_compare <- elig %>%
  mutate(Died = ifelse(HOSP_OUTCM_grp == "Death", "Yes", "No")) %>%
  tbl_summary(
    by      = BMI_missing,
    include = c(AGE, SEX, ANZRODRiskOfDeath, PA_FI_ratio, FRAILTY_cat,
                AP3diag_binary, hosp_type, Died),
```

```r
    label   = list(
      AGE               ~ "Age (years)",
      SEX               ~ "Sex",
      ANZRODRiskOfDeath ~ "ANZROD risk of death (%)",
      PA_FI_ratio       ~ "PaO2/FiO2 ratio (mmHg)",
      FRAILTY_cat       ~ "Clinical Frailty Scale",
      AP3diag_binary    ~ "Admission diagnosis",
      hosp_type         ~ "Hospital type",
      Died              ~ "In-hospital death"
    ),
    statistic = list(all_continuous() ~ "{median} [{p25}, {p75}]"),
    missing   = "no"
  ) %>%
  add_overall(last = TRUE) %>%
  modify_caption("Eligible patients with vs without recorded BMI")

print(tbl_compare)

# ---- 2. Site-level auxiliaries + MICE setup --------------------------------
# Site-level auxiliaries for the imputation: BMI recording rate and mean
# observed BMI, rather than hundreds of site dummies.
site_aux <- elig %>%
  group_by(DSITEID) %>%
  summarise(site_bmi_mean = mean(BMI, na.rm = TRUE),
            site_bmi_rec  = mean(!is.na(BMI)), .groups = "drop")
site_aux$site_bmi_mean[is.nan(site_aux$site_bmi_mean)] <- mean(elig$BMI, na.rm = TRUE)
elig <- left_join(elig, site_aux, by = "DSITEID")

imp_vars <- c("BMI", "AGE_scaled", "SEX", "ANZRODRiskOfDeath_scaled",
              "PA_FI_ratio", "FRAILTY_cat", "AP3diag_binary", "hosp_type",
              "hosp_death", "site_bmi_mean", "site_bmi_rec")
mi_input <- elig[, imp_vars]

ini  <- mice(mi_input, maxit = 0, printFlag = FALSE)
meth <- ini$method;         meth[] <- "";      meth["BMI"] <- "pmm"  # impute BMI only
pred <- ini$predictorMatrix; pred[] <- 0
pred["BMI", setdiff(imp_vars, "BMI")] <- 1                          # outcome included

imp <- mice(mi_input, m = m, method = meth, predictorMatrix = pred,
            printFlag = FALSE, seed = 2392)

# Long form + re-attach site for the random effect (kept out of the imputation model)
long <- complete(imp, "long")
long$DSITEID     <- elig$DSITEID[long$.id]
long$was_imputed <- is.na(elig$BMI)[long$.id]

# ---- 3. Model fit + Rubin pooling helpers ----------------------------------
bmi_breaks <- c(-Inf, 18.5, 25, 30, 35, 40, Inf)
bmi_labs   <- c("Underweight", "Normal", "Pre Obese",
                "Obese Class 1", "Obese Class 2", "Obese Class 3")

fit_one <- function(dat) {
  dat$BMICAT <- cut(dat$BMI, breaks = bmi_breaks, labels = bmi_labs, right = FALSE)
  dat$BMICAT <- relevel(factor(dat$BMICAT), ref = "Normal")
  glmer(hosp_death ~ BMICAT + AGE_scaled + SEX + AP3diag_binary +
          ANZRODRiskOfDeath_scaled + hosp_type + FRAILTY_cat +
          ns(PA_FI_ratio, df = 4) + (1 | DSITEID),
        data = dat, family = binomial,
        control = glmerControl(optimizer = "bobyqa", optCtrl = list(maxfun = 2e5)))
```

```
}

bmi_coefs <- function(mod) {
  s  <- summary(mod)$coefficients
  rn <- grep("^BMICAT", rownames(s), value = TRUE)
  data.frame(term = rn, est = s[rn, "Estimate"], se = s[rn, "Std. Error"],
             row.names = NULL)
}

# Rubin's rules on the log-OR scale, returned as OR + 95% CI
pool_rubin <- function(coef_list, m) {
  terms <- coef_list[[1]]$term
  do.call(rbind, lapply(terms, function(t) {
    e <- sapply(coef_list, function(d) d$est[d$term == t])
    u <- sapply(coef_list, function(d) d$se [d$term == t])^2
    qbar <- mean(e); ubar <- mean(u); b <- var(e)
    se   <- sqrt(ubar + (1 + 1 / m) * b)
    data.frame(term = t, OR = exp(qbar),
               lo = exp(qbar - z * se), hi = exp(qbar + z * se))
  }))
}

fit_across_imps <- function(shift = 0) {
  coef_list <- lapply(seq_len(m), function(k) {
    dat <- long[long$.imp == k, ]
    if (shift != 0) dat$BMI[dat$was_imputed] <- dat$BMI[dat$was_imputed] + shift
    bmi_coefs(fit_one(dat))
  })
  pool_rubin(coef_list, m)
}

# ---- 4. Table S2: complete-case vs MAR imputation (delta = 0) ---------------
mod_cc <- fit_one(elig[!is.na(elig$BMI), ])
cc <- bmi_coefs(mod_cc) %>%
  transmute(term, OR = exp(est), lo = exp(est - z * se), hi = exp(est + z * se))

mar <- fit_across_imps(shift = 0)

panelB <- cc %>%
  transmute(`BMI category` = sub("^BMICAT", "", term),
            `Complete-case OR (95% CI)` = fmt_or(OR, lo, hi)) %>%
  left_join(
    mar %>% transmute(`BMI category` = sub("^BMICAT", "", term),
                      `MAR imputation OR (95% CI)` = fmt_or(OR, lo, hi)),
    by = "BMI category")

cat("\n--- Table S2: complete-case vs MAR imputation ---\n"); print(panelB)

# ---- 5. Table S3: MNAR tipping-point (shift IMPUTED BMI by delta) -----------
cat("\n--- Table S3: tipping-point (shifting imputed BMI by delta kg/m2) ---\n")
tip <- do.call(rbind, lapply(delta_grid, function(d) {
  cat("  fitting delta =", d, "\n")
  out <- fit_across_imps(shift = d)
  out$delta <- d
  out
}))
tip$`BMI category` <- sub("^BMICAT", "", tip$term)
tip$`OR (95% CI)`  <- fmt_or(tip$OR, tip$lo, tip$hi)
tip$crosses_null   <- tip$lo <= 1 & tip$hi >= 1     # CI includes 1
```

```
tip_wide <- tidyr::pivot_wider(
  tip[, c("BMI category", "delta", "OR (95% CI)")],
  names_from = delta, values_from = `OR (95% CI)`,
  names_prefix = "delta=")
cat("\nTipping-point ORs by delta:\n"); print(as.data.frame(tip_wide))
```

5. Primary analysis

5.1 Prepare analytic dataset

```
apd_final <- apd_final %>%
  mutate(
    hosp_death     = factor(ifelse(HOSP_OUTCM_grp == "Death", 1, 0)),
    SEX            = factor(SEX),
    AP3diag_binary = factor(AP3diag_binary),
    hosp_type      = factor(hosp_type),
    FRAILTY_cat    = factor(FRAILTY_cat),
    DSITEID        = factor(DSITEID)
  )

apd_final$AGE_scaled               <- scale(apd_final$AGE)
apd_final$ANZRODRiskOfDeath_scaled <- scale(apd_final$ANZRODRiskOfDeath)

apd_filtered <- apd_final %>%
  filter(NIV_IND != 1 | is.na(NIV_IND)) %>%
  group_by(DSITEID) %>%
  filter(n() > 50) %>%
  ungroup()

apd_filtered$BMICAT <- relevel(factor(apd_filtered$BMICAT), ref = "Normal")
```

5.2 Main model

```
mod_hosp_death_severity <- glmer(
  hosp_death ~ BMICAT + AGE_scaled + SEX + AP3diag_binary +
    ANZRODRiskOfDeath_scaled + hosp_type + FRAILTY_cat +
    ns(PA_FI_ratio, df = 4) + (1 | DSITEID),
  data    = apd_filtered,
  family  = binomial,
  control = glmerControl(optimizer = "bobyqa", optCtrl = list(maxfun = 2e5))
)

summary(mod_hosp_death_severity)
```

5.3 Between-hospital clustering (contributing hospitals and ICC)

```
# Reported in the footnote of Table S6.
n_hosp <- nlevels(droplevels(apd_filtered$DSITEID))
tau2   <- as.numeric(lme4::VarCorr(mod_hosp_death_severity)$DSITEID)
icc    <- tau2 / (tau2 + pi^2 / 3)          # latent-variable (logistic) ICC
```

```r
cat("Contributing hospitals:      ", n_hosp,           "\n")
cat("Between-hospital variance:   ", round(tau2, 3),  "\n")
cat("Intraclass correlation (ICC):", round(icc, 3),   "\n")
```

6. Interaction test: BMI x PA/FI ratio (Table S6)

```r
mod_int_hosp_death <- glmer(
  hosp_death ~ BMICAT * ns(PA_FI_ratio, df = 4) + AGE_scaled + SEX +
    AP3diag_binary + ANZRODRiskOfDeath_scaled + hosp_type + FRAILTY_cat +
    (1 | DSITEID),
  data    = apd_filtered,
  family  = binomial,
  control = glmerControl(optimizer = "bobyqa", optCtrl = list(maxfun = 2e5))
)

anova(mod_hosp_death_severity, mod_int_hosp_death)
```

7. Visualisation: PA/FI ratio and odds of hospital death (Figure 2)

```r
pf_grid <- data.frame(
  PA_FI_ratio              = seq(min(apd_filtered$PA_FI_ratio, na.rm = TRUE),
                                 max(apd_filtered$PA_FI_ratio, na.rm = TRUE),
                                 length.out = 200),
  BMICAT                   = "Normal",
  AGE_scaled               = 0,
  SEX                      = "M",
  AP3diag_binary           = "Other diagnoses",
  ANZRODRiskOfDeath_scaled = 0,
  hosp_type                = "Public Tertiary",
  FRAILTY_cat              = "1-3"
)

ref_pf <- mean(apd_filtered$PA_FI_ratio, na.rm = TRUE)

pf_grid$logodds <- predict(mod_hosp_death_severity,
                           newdata = pf_grid,
                           re.form = NA,
                           type    = "link")

ref_row             <- pf_grid[1, ]
ref_row$PA_FI_ratio <- ref_pf
ref_logodds         <- predict(mod_hosp_death_severity,
                               newdata = ref_row,
                               re.form = NA,
                               type    = "link")

pf_grid$OR <- exp(pf_grid$logodds - ref_logodds)

ggplot(pf_grid, aes(x = PA_FI_ratio, y = OR)) +
  geom_hline(yintercept = 1, linetype = "dashed", color = "gray40") +
  geom_line(color = "#2171B5", linewidth = 1.2) +
  labs(
```

```
    title    = "Odds ratio for hospital death across PA/FI ratio",
    subtitle = paste0("Reference PA/FI = mean = ", round(ref_pf, 0)),
    x        = "PA/FI ratio",
    y        = "Odds ratio"
  ) +
  theme_minimal(base_size = 13)
```

8. COVID-19 sensitivity analysis (Tables S7–S8)

We test effect modification by fitting a BMI × COVID-19 interaction in the model cohort rather than splitting it, so the whole sample feeds a single likelihood-ratio test. The model is otherwise identical to the primary analysis.

8.1 Characteristics by COVID-19 status (Table S8)

```
cat("COVID-19 admissions:  ", sum(apd_final$covid == "COVID-19"),     "\n")
cat("Non-COVID admissions: ", sum(apd_final$covid == "Not COVID-19"), "\n")

tbl_covid <- apd_final %>%
  mutate(Died = ifelse(HOSP_OUTCM_grp == "Death", "Yes", "No")) %>%
  tbl_summary(
    by      = covid,
    include = c(AGE, SEX, ANZRODRiskOfDeath, PA_FI_ratio,
                FRAILTY_cat, BMICAT, Died),
    label   = list(
      AGE               ~ "Age (years)",
      SEX               ~ "Sex",
      ANZRODRiskOfDeath ~ "ANZROD risk of death (%)",
      PA_FI_ratio       ~ "PaO2/FiO2 ratio (mmHg)",
      FRAILTY_cat       ~ "Clinical Frailty Scale",
      BMICAT            ~ "BMI category",
      Died              ~ "In-hospital death"
    ),
    statistic = list(all_continuous() ~ "{median} [{p25}, {p75}]"),
    missing   = "no"
  ) %>%
  add_overall(last = TRUE) %>%
  modify_caption("Characteristics by COVID-19 status (analysis cohort)")

print(as_gt(tbl_covid))
```

8.2 Interaction model, LRT, and stratum-specific odds ratios (Table S7)

```
# Both carry the COVID-19 main effect and differ only by the interaction,
# so the LRT is the test of effect modification.
covariates <- "AGE_scaled + SEX + AP3diag_binary + ANZRODRiskOfDeath_scaled +
               hosp_type + FRAILTY_cat + ns(PA_FI_ratio, df = 4) + (1 | DSITEID)"

mod_covid_main <- glmer(
  as.formula(paste("hosp_death ~ BMICAT + covid +", covariates)),
  data = apd_filtered, family = binomial,
  control = glmerControl(optimizer = "bobyqa", optCtrl = list(maxfun = 2e5))
)
```

```r
mod_covid_int <- glmer(
  as.formula(paste("hosp_death ~ BMICAT * covid +", covariates)),
  data = apd_filtered, family = binomial,
  control = glmerControl(optimizer = "bobyqa", optCtrl = list(maxfun = 2e5))
)

# Likelihood-ratio test for effect modification
lrt <- anova(mod_covid_main, mod_covid_int)
print(lrt)
cat("\nInteraction LRT p-value: ", signif(lrt$`Pr(>Chisq)`[2], 3), "\n\n")

# BMI odds ratios (vs Normal) within each COVID-19 stratum
emm <- emmeans(mod_covid_int, ~ BMICAT | covid)
ctr <- contrast(emm, method = "trt.vs.ctrl", type = "response")  # vs Normal
or_strata <- as.data.frame(summary(ctr, infer = TRUE))

names(or_strata)[grepl("LCL|lower", names(or_strata))] <- "lo"
names(or_strata)[grepl("UCL|upper", names(or_strata))] <- "hi"

or_tbl <- or_strata %>%
  transmute(
    `BMI category`    = gsub(" - .*| / .*", "", contrast),
    `COVID-19 status`= covid,
    `Adjusted OR`     = sprintf("%.2f (%.2f-%.2f)", odds.ratio, lo, hi),
    `p-value`         = signif(p.value, 2)
  )

print(gt(or_tbl) |>
        tab_header(title = "BMI odds ratios vs Normal, by COVID-19 status"))
```

8.3 Forest plot of stratum-specific BMI odds ratios

```r
or_strata <- or_strata %>%
  mutate(bmi = factor(gsub(" - .*| / .*", "", contrast),
                      levels = rev(c("Underweight", "Pre Obese",
                                     "Obese Class 1", "Obese Class 2",
                                     "Obese Class 3"))))

ggplot(or_strata, aes(x = odds.ratio, y = bmi, colour = covid)) +
  geom_vline(xintercept = 1, linetype = "dashed", colour = "gray40") +
  geom_pointrange(aes(xmin = lo, xmax = hi),
                  position = position_dodge(width = 0.5)) +
  scale_x_log10() +
  labs(x = "Adjusted odds ratio (vs Normal BMI)", y = NULL, colour = NULL,
       title = "BMI-mortality association by COVID-19 status") +
  theme_minimal(base_size = 13)
```

9. ECMO recipients: descriptive analysis (Table S10)

```r
# Descriptive only. ECMO_IND does not distinguish veno-venous from veno-arterial
# support, and cell counts are small, so no model is fitted.
ecmo <- apd_final %>%
  filter(ECMO_IND == 1) %>%
  mutate(Died = ifelse(HOSP_OUTCM_grp == "Death", "Yes", "No"))
```

```r
ecmo %>%
  tbl_summary(
    by      = BMICAT,
    include = c(AGE, SEX, ANZRODRiskOfDeath, PA_FI_ratio, FRAILTY_cat,
                AP3diag_binary, INV_HOURS, ICU_HRS, HOSP_HRS, TRACHE_IND, Died),
    label   = list(
      AGE               ~ "Age (years)",
      SEX               ~ "Sex",
      ANZRODRiskOfDeath ~ "ANZROD risk of death (%)",
      PA_FI_ratio       ~ "PaO2/FiO2 ratio (mmHg)",
      FRAILTY_cat       ~ "Clinical Frailty Scale",
      AP3diag_binary    ~ "Admission diagnosis",
      INV_HOURS         ~ "Duration of mechanical ventilation (hours)",
      ICU_HRS           ~ "ICU length of stay (hours)",
      HOSP_HRS          ~ "Hospital length of stay (hours)",
      TRACHE_IND        ~ "Tracheostomy",
      Died              ~ "In-hospital death"
    ),
    statistic = list(all_continuous() ~ "{median} [{p25}, {p75}]"),
    missing   = "no"
  ) %>%
  add_overall(last = TRUE) %>%
  modify_caption("ECMO recipients: characteristics and crude mortality by BMI
category")
```

10. Session information

```r
sessionInfo()
```
