# Supplementary material for: Body mass index and hospital mortality in mechanically ventilated patients with acute hypoxaemic respiratory failure: A retrospective cohort study
Source: Crit Care Resusc. 2026 Sep 8;28(4):100216. doi: 10.1016/j.ccrj.2026.100216 (PMC13579679; doi:10.1016/j.ccrj.2026.100216)
Supplement: Supplementary file 2 [file mmc2.docx]

**Supplementary Material**

**Body Mass Index and Hospital Mortality in Mechanically Ventilated Patients with Acute Hypoxaemic Respiratory Failure: A Retrospective Cohort Study**

**TABLE OF CONTENTS**

**Supplementary Tables**

**Table S1.** Like for like comparison. Baseline characteristics and crude in-hospital mortality of eligible patients with versus without a recorded BMI, restricted to the eligible population.

**Table S2.** Unadjusted (crude) in-hospital mortality by BMI category.

**Table S3.** Characteristics of patients with versus without a recorded Clinical Frailty Scale score.

**Table S4.** Adjusted odds ratios for in-hospital mortality by BMI category: complete-case analysis versus multiple imputation by chained equations under a missing-at-random assumption (40 imputations, pooled by Rubin's rules).

**Table S5.** Missing-not-at-random sensitivity (tipping-point) analysis: adjusted odds ratios across delta shifts of −3 to +3 kg/m² applied to imputed BMI values.

**Table S6.** Quantitative bias assessment. Selection-bias E-values.

**Table S7.** Hierarchical model comparison for the BMI × PaO₂/FiO₂ interaction (AIC, BIC, log-likelihood, degrees of freedom), with the number of contributing hospitals and the between-hospital variance/ICC.

**Table S8.** Adjusted odds ratios for in-hospital mortality by BMI category, stratified by COVID-19 status (COVID-19 sensitivity analysis).

**Table S9.** Baseline characteristics of patients with confirmed COVID-19 versus without, within the analytic cohort.

**Table S10.** Secondary outcomes by BMI category: duration of mechanical ventilation, ICU and hospital length of stay, and ECMO and tracheostomy receipt.

**Table S11.** Characteristics, interventions and crude in-hospital mortality among ECMO recipients by BMI category. ECMO recipients were identified by the APD flag, which does not distinguish veno-venous from veno-arterial support.

**Supplementary Figures**

**Figure S1.** Directed acyclic graph of the assumed causal structure, showing conditioning on intubation (cohort selection) as a collider on the path between BMI and mortality.

**Figure S2.** Unadjusted in-hospital mortality across BMI categories.

Table S1.

Missing data analysis. Like for like comparison. Eligible patients with vs without recorded BMI

Note: Overall = 64,779 (different from analytical cohort) due to complete covariate inclusion criteria.

| **Characteristic** | **BMI missing**  N = 24,510^1^ | **BMI recorded**  N = 40,269^1^ | **Overall**  N = 64,779^1^ |
| --- | --- | --- | --- |
| Age (years) | 60 [46, 71] | 60 [47, 70] | 60 [46, 71] |
| Sex |  |  |  |
| F | 9,522 (39%) | 14,645 (36%) | 24,167 (37%) |
| M | 14,988 (61%) | 25,624 (64%) | 40,612 (63%) |
| ANZROD risk of death (%) | 10 [3, 32] | 10 [3, 31] | 10 [3, 31] |
| PaO2/FiO2 ratio (mmHg) | 178 [122, 238] | 177 [121, 237] | 177 [122, 238] |
| Clinical Frailty Scale |  |  |  |
| 1-3 | 14,612 (60%) | 24,219 (60%) | 38,831 (60%) |
| 4-6 | 9,176 (37%) | 15,021 (37%) | 24,197 (37%) |
| 7-9 | 722 (2.9%) | 1,029 (2.6%) | 1,751 (2.7%) |
| Admission diagnosis |  |  |  |
| Other diagnoses | 19,910 (81%) | 33,027 (82%) | 52,937 (82%) |
| Respiratory diagnoses | 4,600 (19%) | 7,242 (18%) | 11,842 (18%) |
| Hospital type |  |  |  |
| Private | 1,543 (6.3%) | 2,131 (5.3%) | 3,674 (5.7%) |
| Public Metropolitan | 7,570 (31%) | 8,567 (21%) | 16,137 (25%) |
| Public Rural / Regional | 3,930 (16%) | 5,156 (13%) | 9,086 (14%) |
| Public Tertiary | 11,467 (47%) | 24,415 (61%) | 35,882 (55%) |
| In-hospital death | 5,430 (22%) | 8,266 (21%) | 13,696 (21%) |
| ^1^Median [Q1, Q3]; n (%) | | | |

## Table S2.

## Unadjusted Hospital Mortality by BMI Category

Crude hospital mortality rates stratified by body mass index category, before adjustment for confounders.

| **BMI Category** | **N** | **Deaths (n)** | **Mortality (%)** |
| --- | --- | --- | --- |
| Underweight (<18.5 kg/m²) | 719 | 176 | 24.5 |
| Healthy weight (18.5-24.9 kg/m²) | 10,112 | 2,277 | 22.7 |
| Pre-obese (25-29.9 kg/m²) | 12,177 | 2,581 | 21.4 |
| Obese Class 1 (30-34.9 kg/m²) | 8,283 | 1,637 | 20.1 |
| Obese Class 2 (35-39.9 kg/m²) | 4,479 | 823 | 18.8 |
| Obese Class 3 (≥40 kg/m²) | 4,582 | 772 | 17.2 |
| **Overall** | **40,352** | **8,266** | **21** |

BMI = Body Mass Index.

## Table S3.

## Patients with and without Clinical Frailty Score recorded.

| **Characteristic** | **Patients with CFS**  N = 40,352^1^ | **Patients without CFS**  N = 153,637^1^ | **Overall**  N = 193,989^1^ |
| --- | --- | --- | --- |
| Age | 60 [47, 70] | 66 [52, 75] | 64 [51, 74] |
| Gender |  |  |  |
| F | 14,673 (36%) | 63,395 (41%) | 78,068 (40%) |
| M | 25,679 (64%) | 90,147 (59%) | 115,826 (60%) |
| BMI Category |  |  |  |
| Underweight | 719 (1.8%) | 2,131 (2.9%) | 2,850 (2.5%) |
| Healthy weight. | 10,112 (25%) | 20,367 (28%) | 30,479 (27%) |
| Pre Obese | 12,177 (30%) | 22,733 (31%) | 34,910 (31%) |
| Obese Class 1 | 8,283 (21%) | 14,386 (20%) | 22,669 (20%) |
| Obese Class 2 | 4,479 (11%) | 6,570 (9.1%) | 11,049 (9.8%) |
| Obese Class 3 | 4,582 (11%) | 6,166 (8.5%) | 10,748 (9.5%) |
| ICU Diagnosis |  |  |  |
| Other diagnoses | 33,099 (82%) | 140,477 (92%) | 173,576 (90%) |
| Respiratory diagnoses | 7,253 (18%) | 12,519 (8.2%) | 19,772 (10%) |
| ANZROD Risk of Death score (%) | 10 [3, 31] | 1 [0, 6] | 2 [1, 11] |
| Hospital type |  |  |  |
| Private | 2,141 (5.3%) | 55,281 (36%) | 57,422 (30%) |
| Public Metropolitan | 8,579 (21%) | 6,607 (4.3%) | 15,186 (7.8%) |
| Public Rural / Regional | 5,161 (13%) | 20,353 (13%) | 25,514 (13%) |
| Public Tertiary | 24,471 (61%) | 71,396 (46%) | 95,867 (49%) |
| Hospital Outcome |  |  |  |
| Death | 8,266 (21%) | 12,379 (8.1%) | 20,645 (11%) |
| Home | 20,309 (50%) | 109,993 (72%) | 130,302 (67%) |
| Inter-hospital transfer | 6,442 (16%) | 14,827 (9.7%) | 21,269 (11%) |
| Nursing home/Chronic care/Palliative care | 613 (1.5%) | 2,545 (1.7%) | 3,158 (1.6%) |
| Other | 1,117 (2.8%) | 2,532 (1.7%) | 3,649 (1.9%) |
| Rehabilitation | 3,522 (8.7%) | 10,592 (6.9%) | 14,114 (7.3%) |
| Duration of mechanical ventilation (hours) | 51 [18, 138] | 0 [0, 17] | 13 [0, 63] |
| ICU length of stay (hours) | 113 [51, 231] | 43 [22, 78] | 48 [23, 101] |
| Hospital length of stay (hours) | 283 [134, 546] | 196 [105, 367] | 212 [111, 405] |
| Receipt of a tracheostomy | 2,106 (5.3%) | 1,811 (1.6%) | 3,917 (2.6%) |
| Receipt of ECMO support | 862 (2.2%) | 275 (0.2%) | 1,137 (0.7%) |
| ^1^Median [Q1, Q3]; n (%) | | | |

## Table S4.

**BMI Missing data analysis.**

BMI and hospital mortality: Complete-case vs Missing At Random imputation

| **BMI category** | **Complete-case (Table 2) OR (95% CI)** | **Multiple imputation OR (95% CI)** |  |
| --- | --- | --- | --- |
| Underweight | 1.10 (0.90–1.36) | 1.05 (0.86–1.29) |  |
| Healthy weight | 1.00 (reference) | 1.00 (reference) |  |
| Pre Obese | 0.92 (0.85–0.99) | 0.95 (0.88–1.02) |  |
| Obese Class 1 | 0.85 (0.78–0.93) | 0.89 (0.82–0.97) |  |
| Obese Class 2 | 0.88 (0.79–0.97) | 0.90 (0.82–0.99) |  |
| Obese Class 3 | 0.92 (0.82–1.02) | 0.91 (0.82–1.01) |  |
| The complete-case column is the primary model reported in Table 2. Multiple imputation: 40 imputations of BMI by predictive mean matching (outcome included), pooled by Rubin's rules, on the same cohort with BMI-missing patients retained. | | | |

## MAR = Missing at random, OR = Odds Ratio, CI = Confidence Interval.

## Table S5.

## Missing Not At Random tipping point analysis.

MNAR tipping-point: adjusted OR (vs Normal) by delta shift (kg/m2) applied to imputed BMI

| **BMI category** | **δ=-3** | **δ=-2** | **δ=-1** | **δ=0** | **δ=1** | **δ=2** | **δ=3** |
| --- | --- | --- | --- | --- | --- | --- | --- |
| Underweight | 1.03 (0.88–1.21) | 1.05 (0.88–1.25) | 1.05 (0.87–1.26) | 1.05 (0.86–1.29) | 1.07 (0.87–1.32) | 1.09 (0.88–1.34) | 1.09 (0.88–1.35) |
| Pre Obese | 0.93 (0.86–1.00) | 0.94 (0.87–1.01) | 0.94 (0.88–1.01) | 0.95 (0.88–1.02) | 0.95 (0.89–1.02) | 0.96 (0.89–1.04) | 0.96 (0.89–1.04) |
| Obese Class 1 | 0.87 (0.80–0.94) | 0.88 (0.81–0.95) | 0.89 (0.82–0.96) | 0.89 (0.82–0.97) | 0.91 (0.84–0.99) | 0.92 (0.85–1.00) | 0.93 (0.86–1.01) |
| Obese Class 2 | 0.87 (0.79–0.96) | 0.88 (0.80–0.97) | 0.89 (0.81–0.98) | 0.90 (0.82–0.99) | 0.91 (0.83–1.00) | 0.93 (0.84–1.02) | 0.94 (0.85–1.04) |
| Obese Class 3 | 0.89 (0.80–0.99) | 0.90 (0.81–1.00) | 0.90 (0.81–1.00) | 0.91 (0.82–1.01) | 0.91 (0.83–1.01) | 0.92 (0.83–1.02) | 0.93 (0.84–1.03) |
| *Adjusted odds ratios (vs Normal) under multiple imputation with imputed BMI shifted by δ kg/m² (MNAR sensitivity). δ=0 is the primary imputation result. Reference: Normal BMI.*  *Estimates are from the multiple-imputation analysis; the δ=0 column corresponds to the primary imputation result in Supplementary Table S4. These differ from the complete-case estimates in Table 2 because imputation retains patients with missing BMI."* | | | | | | | |

## Table S6.

## Quantitative bias assessment output. Selection-bias E-values

| **BMI category** | **OR (95% CI)** | **E-value (point)** | **E-value (CI)** |
| --- | --- | --- | --- |
| Underweight | 1.10 (0.90–1.36) | 1.28 | 1.00 |
| Pre Obese | 0.92 (0.85–0.99) | 1.26 | 1.07 |
| Obese Class 1 | 0.85 (0.78–0.93) | 1.38 | 1.23 |
| Obese Class 2 | 0.88 (0.79–0.97) | 1.34 | 1.13 |
| Obese Class 3 | 0.92 (0.82–1.02) | 1.26 | 1.00 |
| *Selection-bias E-values (Smith & VanderWeele, 2019), computed for the selected population. Minimum association (risk-ratio scale) that unmeasured determinants of intubation would need with both BMI and mortality to move the estimate (and CI limit) to 1. Reference: Normal BMI.* | | | |

## Table S7.

## Model Comparison for BMI × Respiratory Failure Severity Interaction

Comparison of hierarchical logistic regression models with and without interaction between BMI category and PaO₂/FiO₂ ratio (modelled using restricted cubic splines, 4 degrees of freedom).

| Model | Parameters | AIC | BIC | Log-likelihood | df |
| --- | --- | --- | --- | --- | --- |
| Main effects only | 20 | 30,225 | 30,396 | -15,092 | — |
| With BMI × PaO₂/FiO₂ interaction | 40 | 30,235 | 30,578 | -15,078 | 20 |
| Likelihood ratio test | χ² = 29.7 | df = 20 | p = 0.075 |  |  |

AIC = Akaike Information Criterion; BIC = Bayesian Information Criterion; df = degrees of freedom; PaO₂/FiO₂ = ratio of arterial oxygen partial pressure to fractional inspired oxygen; BMI = Body Mass Index. The main effects model was selected based on better parsimony (lower AIC/BIC)

The model included **174 contributing hospitals**. **The between-hospital variance in the random intercept was 0.06**, with an **intraclass correlation coefficient of 0.02**.

Table S8.

BMI category and in-hospital mortality, by COVID-19 status. Adjusted for age, sex, admission diagnosis, ANZROD, hospital type, Clinical Frailty Scale and PaO2/FiO2 (natural cubic spline), with site as a random effect.

|  | Adjusted OR (95% CI) vs Normal BMI | |
| --- | --- | --- |
| **BMI category** | **Not COVID-19** | **COVID-19 Positive** |
| Normal | 1.00 (reference) | 1.00 (reference) |
| Underweight | 1.13 (0.86-1.48) | 0.93 (0.23-3.75) |
| Pre Obese | 0.91 (0.82-1.00) | 1.23 (0.75-2.01) |
| Obese Class 1 | 0.83 (0.74-0.92) | 1.17 (0.67-2.03) |
| Obese Class 2 | 0.84 (0.73-0.96) | 1.12 (0.58-2.16) |
| Obese Class 3 | 0.86 (0.75-0.99) | 1.13 (0.60-2.15) |
| Interaction (BMI category × COVID-19) likelihood-ratio test: χ² = 4.00, df = 5, p = 0.55. | | |
| Main-effects model AIC = 30913; interaction model AIC = 30919. | | |

**Table S9.**

Key metrics by COVID-19 status (analysis cohort)

| **Characteristic** | **Not COVID-19**  N = 39,061^1^ | **COVID-19 Positive**  N = 1,291^1^ | **Overall**  N = 40,352^1^ |
| --- | --- | --- | --- |
| **Age (years)** | 60 [46, 70] | 62 [50, 71] | 60 [47, 70] |
| **Sex** |  |  |  |
| F | 14,216 (36%) | 457 (35%) | 14,673 (36%) |
| M | 24,845 (64%) | 834 (65%) | 25,679 (64%) |
| **ANZROD risk of death (%)** | 10 [3, 31] | 15 [6, 34] | 10 [3, 31] |
| **PaO2/FiO2 ratio (mmHg)** | 178 [122, 238] | 149 [100, 210] | 177 [121, 237] |
| **Clinical Frailty Scale** |  |  |  |
| 1-3 | 23,525 (60%) | 737 (57%) | 24,262 (60%) |
| 4-6 | 14,540 (37%) | 516 (40%) | 15,056 (37%) |
| 7-9 | 996 (2.5%) | 38 (2.9%) | 1,034 (2.6%) |
| **BMI category** |  |  |  |
| Normal | 9,792 (25%) | 320 (25%) | 10,112 (25%) |
| Underweight | 694 (1.8%) | 25 (1.9%) | 719 (1.8%) |
| Pre Obese | 11,794 (30%) | 383 (30%) | 12,177 (30%) |
| Obese Class 1 | 8,032 (21%) | 251 (19%) | 8,283 (21%) |
| Obese Class 2 | 4,330 (11%) | 149 (12%) | 4,479 (11%) |
| Obese Class 3 | 4,419 (11%) | 163 (13%) | 4,582 (11%) |
| **In-hospital death** | 7,907 (20%) | 359 (28%) | 8,266 (21%) |
| ^1^Median [Q1, Q3]; n (%) | | | |

## Table S10.

## Secondary Outcomes by BMI Category

Duration of mechanical ventilation, ICU and hospital length of stay, ECMO and tracheostomy receipt.

| **BMI Category** | **Total number** | **Mechanical Ventilation (hrs) [IQR]** | **ICU LOS (hrs)**  **[IQR]** | **Hosp LOS (hrs)**  **[IQR]** | **ECMO n (%)** | **Trach n (%)** |
| --- | --- | --- | --- | --- | --- | --- |
| Underweight | 719 | 50 [19, 125] | 117 [58, 231] | 285 [126, 568] | 7 (1.0) | 38 (5.3) |
| Healthy weight | 10,112 | 52 [18, 136] | 112 [52, 225] | 282 [129, 553] | 237 (2.3) | 566 (5.7) |
| Pre-obese | 12,177 | 51 [18, 137] | 113 [51, 231] | 281 [133, 549] | 276 (2.3) | 660 (5.5) |
| Obese Class 1 | 8,283 | 50 [17, 136] | 112 [50, 228] | 280 [134, 526] | 173 (2.1) | 404 (5.0) |
| Obese Class 2 | 4,479 | 53 [17, 149] | 116 [52, 239] | 288 [139, 544] | 95 (2.1) | 225 (5.1) |
| Obese Class 3 | 4,582 | 52 [18, 141] | 114 [52, 238] | 287 [147, 554] | 71 (1.5) | 216 (4.8) |

MV = Mechanical Ventilation; ICU = Intensive Care Unit; LOS = Length of Stay; Hosp = Hospital; ECMO = Extracorporeal Membrane Oxygenation; Trach = Tracheostomy; BMI = Body Mass Index. Hospital LOS and tracheostomy data to be completed.

Table S11.

ECMO recipients: characteristics, interventions and outcomes by BMI category

| **Characteristic** | **Healthy weight**  N = 237^1^ | **Underweight**  N = 7^1^ | **Pre Obese**  N = 278^1^ | **Obese Class 1**  N = 174^1^ | **Obese Class 2**  N = 95^1^ | **Obese Class 3**  N = 71^1^ | **Overall**  N = 862^1^ |
| --- | --- | --- | --- | --- | --- | --- | --- |
| **Age (years)** | 48 [36, 59] | 38 [21, 53] | 54 [43, 62] | 53 [44, 61] | 51 [45, 60] | 47 [35, 55] | 52 [41, 60] |
| **Sex** |  |  |  |  |  |  |  |
| F | 78 (33%) | 3 (43%) | 66 (24%) | 47 (27%) | 31 (33%) | 33 (46%) | 258 (30%) |
| **ANZROD risk of death (%)** | 35 [14, 59] | 39 [32, 86] | 31 [12, 62] | 34 [13, 63] | 24 [8, 60] | 14 [5, 56] | 31 [12, 60] |
| **PaO2/FiO2 ratio (mmHg)** | 114 [79, 165] | 105 [78, 126] | 100 [71, 147] | 96 [72, 133] | 89 [67, 127] | 79 [64, 113] | 99 [72, 145] |
| **Clinical Frailty Scale** |  |  |  |  |  |  |  |
| 1-3 | 187 (79%) | 4 (57%) | 211 (76%) | 128 (74%) | 73 (77%) | 45 (63%) | 648 (75%) |
| 4-6 | 45 (19%) | 3 (43%) | 65 (23%) | 46 (26%) | 22 (23%) | 26 (37%) | 207 (24%) |
| 7-9 | 5 (2.1%) | 0 (0%) | 2 (0.7%) | 0 (0%) | 0 (0%) | 0 (0%) | 7 (0.8%) |
| **Admission diagnosis** |  |  |  |  |  |  |  |
| Other diagnoses | 184 (78%) | 6 (86%) | 217 (78%) | 130 (75%) | 68 (72%) | 41 (58%) | 646 (75%) |
| Respiratory diagnoses | 53 (22%) | 1 (14%) | 61 (22%) | 44 (25%) | 27 (28%) | 30 (42%) | 216 (25%) |
| **Duration of mechanical ventilation (hours)** | 205 [105, 393] | 239 [75, 993] | 239 [119, 495] | 233 [92, 464] | 214 [102, 385] | 334 [130, 635] | 230 [105, 457] |
| **ICU length of stay (hours)** | 313 [171, 607] | 323 [164, 1,168] | 339 [159, 669] | 285 [103, 619] | 291 [162, 577] | 448 [141, 755] | 323 [154, 642] |
| **Hospital length of stay (hours)** | 503 [233, 919] | 518 [164, 1,532] | 481 [217, 900] | 418 [140, 840] | 494 [232, 1,038] | 717 [200, 995] | 478 [207, 928] |
| **Tracheostomy receipt** | 50 (21%) | 2 (29%) | 52 (19%) | 36 (21%) | 17 (18%) | 18 (25%) | 175 (20%) |
| **In-hospital death** | 62 (26%) | 3 (43%) | 92 (33%) | 74 (43%) | 28 (29%) | 16 (23%) | 275 (32%) |
| ^1^Median [Q1, Q3]; n (%) | | | | | | | |
| Descriptive data only. ECMO recipients identified by the APD ECMO flag, which does not distinguish veno-venous from veno-arterial ECMO. | | | | | | | |

# Supplementary Figures

## Figure S1. Directed Acyclic Graph

Directed acyclic graph (DAG) illustrating assumed causal relationships between body mass index, potential confounders, and hospital mortality. Variables in the adjustment set: ANZROD score, age, sex, ICU admission diagnosis category, hospital type, and Clinical Frailty Scale. Intubation node included as a square to represent a conditional variable. The recognition of unmeasured or unknown variables influencing the Intubation node included as a dashed circular node.

## Figure S2. Unadjusted mortality by BMI category graph
